# Encouraging performance monitoring promotes proactive control in children

**DOI:** 10.1111/desc.12861

**Published:** 2019-06-14

**Authors:** Lauren V. Hadley, Frantzy Acluche, Nicolas Chevalier

**Affiliations:** ^1^ University of Nottingham Glasgow UK; ^2^ East Michigan University Ypsilanti Michigan; ^3^ University of Edinburgh Edinburgh UK

**Keywords:** children, cognitive control, event‐related potentials, inhibition, performance monitoring, proactive control

## Abstract

Monitoring progression towards one's goals is essential for efficient cognitive control. Immature performance monitoring may contribute to suboptimal cognitive control engagement in childhood, potentially explaining why children engage control reactively even when proactive control would be more effective. This study investigated whether encouraging children to actively monitor their performance results in more mature control engagement. Electroencephalography data were collected while children and adults performed a flanker task in three conditions in which they were provided no feedback, standard feedback, or were asked to estimate their own feedback. Both age groups accurately estimated their own feedback. Critically, feedback estimation promoted online performance monitoring and proactive engagement of attention and inhibition during the flanker period in children. These findings indicate that proactive control engagement in childhood can be effectively supported by encouraging performance monitoring.

## Graphical abstract

We tested whether children show inefficient control engagement due to difficulty monitoring their performance. When 5‐7‐year‐olds had to choose appropriate feedback for their performance on a flanker task, they showed increased monitoring and proactive control compared to when they received standard or no feedback. We argue that metacognitive reflection is critical for efficient control engagement.

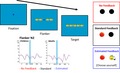

**Research Highlights**

- We investigated whether immature performance monitoring contributes to inefficient cognitive control engagement in 5‐ to 7‐year‐old children.
- Cognitive control and attention were measured during a flanker task in which participants received standard feedback, no feedback, or estimated their own feedback.
- Children showed evidence of monitoring when required to estimate feedback and also showed increased proactive control through N2 amplitude.
- As standard feedback did not lead to monitoring, metacognitive reflection may be critical for efficient cognitive control engagement.

## 1 INTRODUCTION

To reach a goal, be it a score in a test or a time in a race, it is important to identify whether current performance is on track. Performance monitoring can be conceptualized as a feedback loop that computes the deviation between the predicted outcome of an action and its actual implementation (Ullsperger, Danielmeier, & Jocham, 2014). When an individual is making poor progress towards their goal, the large discrepancy between prediction and reality can be used to make the necessary adjustments for success. These adjustments can involve changing the way one engages cognitive control, which refers to the goal‐directed regulation of attention, thoughts and actions. This study tested whether children engage control more proactively when encouraged to monitor performance.

Although feedback processing is refined during childhood, even young children can process feedback about their performance (Mai et al., 2011; Peters, Koolschijn, Crone, Van Duijvenvoorde, & Raijmakers, 2014). However, children do not show mature online performance monitoring (i.e. before feedback is provided) until adolescence (Crone, Somsen, Zanolie, & Van der Molen, 2006). Several event‐related potentials (ERPs) differentiate correct from erroneous responses during or immediately after a response, including the correct‐response positivity (CRP), a positive deflection enhanced during the production of a correct response, and the error‐related negativity (ERN), a negative deflection enhanced following the production of an error (Davies, Segalowitz, & Gavin, 2004). A reduced ERN in children relative to adults (DuPuis et al., 2015; Lo, 2018; Tamnes, Walhovd, Torstveit, Sells, & Fjell, 2013) suggests children either do not generate such strong performance expectations or do not compare these with actual performance (Falkenstein, Hoormann, Christ, & Hohnsbein, 2000). As performance monitoring is supported by anterior and posterior cingulate cortices (Tamnes et al., 2013), developmental improvements in performance monitoring may reflect increasing functional connectivity between these regions and the lateral prefrontal cortex during childhood (Kelly et al., 2009; Marek, Hwang, Foran, Hallquist, & Luna, 2015).

Immature performance monitoring may contribute to suboptimal cognitive control engagement in early childhood, as it may provide poor information about how to engage control to meet task demands. Indeed, young children engage cognitive control in a more rigid fashion than adults. When upcoming task demands are known, control can be engaged proactively by actively anticipating and preparing for the upcoming task, hence minimizing how much conflict will be experienced (Braver, 2012). However, adults flexibly modulate their mode of control according to context, making use of proactive preparation when possible (Braver, Paxton, Locke, & Barch, 2009; Chiew & Braver, 2017; Church, Bunge, Petersen, & Schlaggar, 2017; Giesen, Weissmann, & Rothermund, 2018), young children over‐rely on reactive control, that is, they engage control as needed in the moment to resolve conflict that has already arisen (Chatham, Frank, & Munakata, 2009; Chevalier, James, Wiebe, Nelson, & Espy, 2014; Chevalier, Martis, Curran, & Munakata, 2015; Lucenet & Blaye, 2014; Munakata, Snyder, & Chatham, 2012; Voigt et al., 2014). Interestingly, young children do not readily engage proactive control though they are already capable of this control mode and perform better when engaging it (Chevalier & Blaye, 2016; Chevalier et al., 2015). For instance, while 10 year olds engage control proactively whenever possible, 5 year olds only engage control proactively if reactive control is made harder (Chevalier et al., 2015; see also Elke & Wiebe, 2017).

This study tested the hypothesis that improvements in performance monitoring drive increasingly flexible control engagement, including greater reliance on proactive control. Specifically, performance monitoring may support detection that reactive control is suboptimal (in situations where proactive control would be more efficient) and signal the need to engage control proactively instead. If so, encouraging children to monitor their performance should enhance proactive control engagement. This hypothesis was tested in 5‐ to 7 year olds, as children this age are already capable of proactive control but often do not engage it spontaneously, and should be especially likely to benefit from encouragement to monitor performance. Children's performance was compared to that of adults who are efficient proactive control users and thus less likely to vary proactive control engagement as a function of performance monitoring incentive.

Participants completed the flanker task in which a central target is surrounded by either congruent (⇦⇦⇦⇦⇦) or incongruent (⇦⇦⇨⇦⇦) distractors. In incongruent trials, the prepotent response primed by the flankers must be withheld, which is associated with slower and less accurate responses in children and adults (Checa, Castellanos, Abundis‐Gutiérrez, & Rosario Rueda, 2014; Rueda et al., 2004). We presented flankers ahead of the central target, so that participants could either proactively direct attention to the fixation cross (where the target would subsequently be presented) and inhibit the flankers before target onset in order to minimize conflict when subsequently processing the target, or reactively resolve flanker‐related conflict after target onset. Electroencephalography (EEG) data were collected during three conditions with increasing incentive to monitor performance. After each trial, participants either received no feedback, standard (accurate) feedback, or were asked to estimate the feedback they should have received. Although standard feedback should help participants focus on performance monitoring, it may not constitute as strong an incentive to actively monitor performance as asking participants to overtly estimate their feedback.

As no overt response is expected before target onset, proactive attentional directing and inhibition of flankers were measured through two flanker‐locked ERPs: the P1, which occurs 100–130 ms after the presentation of a visual stimulus and indicates enhanced attentional directing (Hillyard, Vogel, & Luck, 1998; Luck, Fan, & Hillyard, 1993), and the anterior N2, which occurs 250–300 ms after the presentation of the to‐be‐suppressed information and is enhanced when irrelevant information must be inhibited or a response withheld (Folstein & Van Petten, 2007). Performance monitoring was examined through the CRP, which peaks around the time of a response and is enhanced when that response is correct (Davies et al., 2004). Increasing performance monitoring across the no‐feedback, standard‐feedback, and feedback–estimation conditions should be evidenced in children by increasing CRP across conditions. This should not occur in adults, who should actively monitor performance regardless of condition. Critically, if increasing performance monitoring leads to greater proactive control engagement, flanker‐locked P1 and N2 should increase with the incentive to monitor performance across conditions in children, but not in adults who should engage proactive control in all conditions.

## 2 METHOD

### 2.1 Participants

Study participants included 27 children (*M*_age_ = 6 years;1 month, range = 5;0–7;5, *SD *= 0.75 years, 9 female) and 30 adults (*M*_age_ = 24 years, range = 19–31 years, *SD* = 3 years, 22 female). An additional two children were recruited but excluded for noisy EEG data (as detailed below) and an additional two adults were excluded due to technical errors. Parental consent was obtained for all child participants. Children received an age‐appropriate toy and certificate of participation, and adults (participants or parents) received £10 compensation.

### 2.2 Materials and procedure

**Figure 1:**
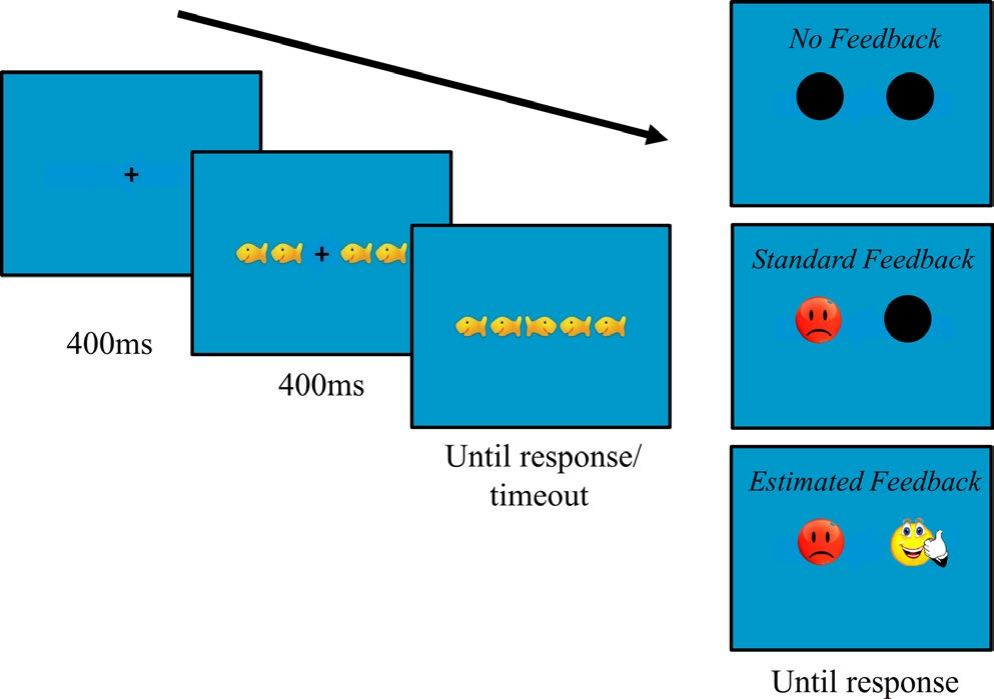
The fish game. The target fish (centre) was oriented in the same direction as the flankers in congruent trials or in the opposite direction on incongruent. On each trial, participants had to indicate the orientation of the target fish

Trained experimenters tested all participants individually in a single session lasting 1.5 hr. After fitting the EEG cap, all participants completed a flanker task in three conditions. The flanker task, run with E‐Prime 2 (Psychology Software Tools, Pittsburgh, PA), required attending to the central target while ignoring distractors (Figure 1). Each trial started with a 400 ms fixation cross, followed by four flanker fish (two on each side of the fixation cross) for another 400 ms. The fixation cross was then replaced with the target fish. On congruent trials, the target fish faced the same direction as the flankers, whereas on incongruent trials, it faced the opposite direction. Congruent and incongruent trials were randomly selected but equally frequent. Participants were instructed to respond to the direction of the target as quickly and accurately as possible by pressing the left or right arrow key on a keyboard. They then saw a different feedback screen in each condition, and they responded to this screen again using the arrow keys. When the target screen was presented, if a response was not made within the individually determined time limit (see below), an additional blank screen was presented for 1 s prior to the feedback screen.

All participants completed standard, no, and estimated feedback conditions (order counterbalanced). Feedback corresponded to a smile if the response was correct, a frown if it was incorrect, or a clock if no response was entered within the participant's adaptive time limit. In the standard feedback condition, the feedback screen showed the accurate feedback beside a black circle (side counterbalanced). In the no feedback condition, the feedback screen showed two black circles. Participants could press the left or right arrow key to continue. In the estimated feedback condition, two different forms of feedback were presented – the accurate feedback and an alternative (side counterbalanced). As the alternative feedback, the two possibilities that remained when the correct feedback was removed were presented with equal probability and random selection. Participants indicated which of the two feedback possibilities they thought was accurate using the arrow keys to continue.

Each condition began with 36 practice trials. The first 28 had a time limit of 3,000 ms, followed by eight trials in which a response time (RT) limit was set according to the initial 28 trials (1.25 × mean RT) in order to familiarize participants to the new time limit before starting test trials. RT limits were subsequently adapted to the participant's performance using a staircase procedure to ensure that the task was of equal difficulty for every participant (decreasing by 50 ms when the response was correct and in time, increasing by 50 ms when the response was incorrect or too late). As each condition included 96 experimental trials, participants completed 288 experimental trials in total.

### 2.3 Data processing

#### 2.3.1 Behavioural

RTs were analysed for correct responses only. Values lower than 200 ms, and greater than each participant's *M* + 3 *SD*, were removed (total 5.8% of trials). All analyses were conducted on log‐transformed RTs to correct for skew. As conditions were blocked, and the adaptive staircase procedure was applied in each block, accuracy data were not analysed due to purposefully low accuracy rates (adult estimated = 50.3% [13.6], adult standard = 50.1% [12.9], adult no feedback = 50.1% [10.6], child estimated = 50.3% [12.3], child standard = 50.9% [13.3], child no feedback = 51.2% [12.3]).

#### 2.3.2 Event‐related potentials

EEG data were recorded at 512 Hz sampling rate using a BioSemi ActiveTwo system with 64 channels, and were processed offline using Brain Vision Analyser 2.1. Impedances were kept under 50 kΩ. Raw data were first filtered using a low cutoff of 0.1 Hz with a roll‐off of 12 dB/octave and a high cut‐off of 30 Hz with a roll‐off of 12 dB/octave. Data were then inspected with a maximal allowed voltage step of 30 μV/ms, a maximal allowed absolute difference of 200 μV within 200 ms, and threshold amplitudes of −200 μV and +200 μV. Any electrodes marked bad for 20% or more of the recording were interpolated by spherical splines (*M* = 3.19, *SD* = 4.27 for children, *M* = 0.75, *SD* = 1.59 for adults). If more than 15 electrodes had been marked bad, the participant was removed (two children). Electrodes were referenced to the average of all electrodes and segmented. Segments underwent ocular correction (Grattton & Coles algorithm) and artefact rejection (using the criteria previously used for data inspection, and an interval beginning 200 ms before the artefact and ending 200 ms after). After baseline correction, the channels and windows used for each ERPs were determined by visual inspection. Amplitude was averaged across each window. Participants with <10 good segments in any experimental cell were removed from the relevant analysis (number of segments per condition specified below).

##### Response‐locked analyses

Segments from −300 ms to 500 ms were extracted, and the 300–200 ms window prior to the response was used as baseline. Given the low number of errors in this task (as opposed to missed responses), performance monitoring was assessed through the CRP. In adults, the CRP was maximal over central channels and therefore analysed between 10–60 ms at Cz (*M* = 47 segments/cell), whereas in children it was slightly delayed and frontally located and hence analysed between 20–70 ms at Fp1 (*M* = 38 segments/cell).

##### Flanker‐locked analyses

Segments from −100 to 800 ms were extracted, and the 100 ms window prior to flanker onset were used as baseline. Proactive control engagement was assessed by the P1 and N2, markers of early attention and inhibition, respectively. P1 was analysed between 110–130 ms and averaged across O1 and O2 in both adults and children. N2 was analysed at FCz between 260–310 ms in adults (*M* = 94 segments/cell) and 300–350 ms in children (*M* = 76 segments/cell).

##### Target‐locked analyses

Segments from −100 to 800 ms were extracted, and the 100 ms window prior to target onset were used as baseline. Reactive control engagement was assessed through the P1 and N2. P1 was analysed between 100–120 ms and averaged across O1 and O2 in both adults and children. N2 was analysed at FCz between 220–270 ms in adults (*M* = 47 segments/cell) and 320–370 ms in children (*M* = 39 segments/cell).

### 2.4 Data analysis

Analyses were conducted in R using the package lme4 (Bates, Maechler, Bolker, & Walker, 2014) and lmerTest (Kuznetsova, Brockhoff, & Christensen, 2015). Linear mixed effect models included full fixed factor specification, and the maximal random factor specification that would converge. When random factor specification needed to be simplified for convergence, interactions then factors were progressively removed until convergence was achieved. In the CRP model, fixed effects comprised accuracy, condition, age group, and all possible interactions, with random effects of accuracy and condition only. In the flanker model, fixed effects comprised condition, age group, and their interaction, with a minimal random effects structure. Finally, in the target model, fixed effects comprised condition, congruence, age group, and all possible interactions, with random effects of condition and congruence only. Significant predictors from the model are reported using ANOVA with degrees of freedom determined using the Satterthwaite method. Subsequent pairwise comparisons were performed using the package emmeans (Lenth, 2018), with Tukey adjustment.

## 3 RESULTS

### 3.1 Did RT vary across conditions?

**Figure 2:**
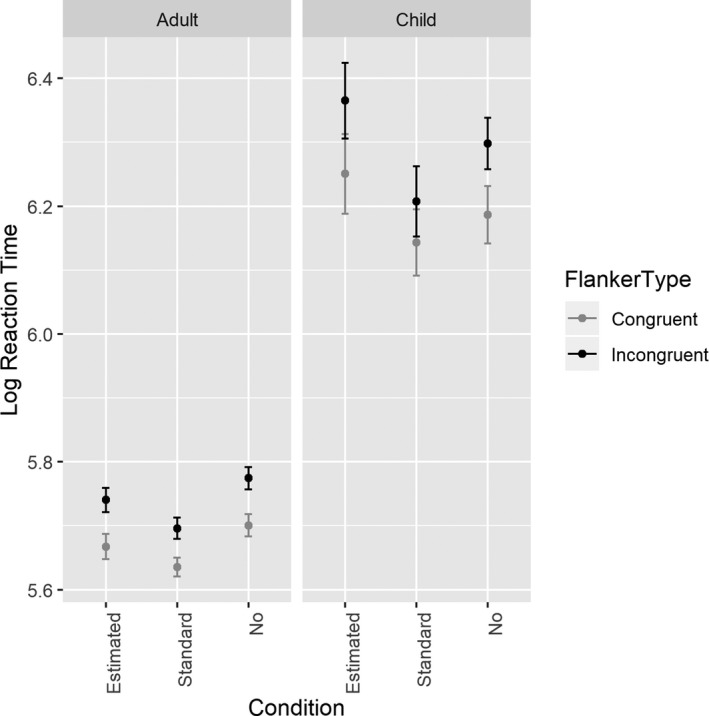
Log transformed reaction times for adults and children by condition and congruence with bars indicating standard error. Responses were slower for incongruent than congruent trials, and children were slower than adults. Children responded more slowly when required to estimate feedback than in either other condition, while adults were slower when provided no feedback compared to standard feedback

RTs were predicted by congruence, *F*(1, 55) = 97.88, *p* < 0.001, age group, *F*(1, 55) = 129.51, *p* < 0.001, condition, *F*(2, 55) = 11.55, *p* < 0.001, as well as the interaction of condition and age group, *F*(2, 55) = 4.92, *p* = 0.012, and condition and congruence, *F*(2, 110) = 3.18, *p* = 0.045 (Figure 2). Adults were slower when they received no feedback than when they received standard feedback, *t*(55) = −2.67, *p *= 0.026. Children, on the other hand, were slower when required to estimate feedback than when they either received no feedback, *t*(55) = 2.49, *p* = 0.041, or received standard feedback *t*(55) = 4.97, *p* < 0.001. Children were also marginally slower when provided no feedback than when provided standard feedback, *t*(55) = −2.34, *p* = 0.058. No other comparisons were significant, *p*s > 0.292. The congruence effect was significant in every condition, *p*s < 0.001, though the difference between congruent and incongruent trials was marginally smaller in the standard condition than either the estimated condition, *t*(110) = 2.23, *p *= 0.071, or the no feedback condition, *t*(110) = −2.14, *p *= 0.087.

### 3.2 Did participants correctly estimate their performance in the estimated feedback condition?

Although adults estimated feedback more accurately than children (adults_m_ = 77%, children_m_ = 65%), *F*(1, 55) = 19.70, *p* < 0.001, both age groups were more accurate at estimating feedback than chance (one‐sample Wilcoxon signed‐rank test adults: *Z *= 4.78, *p* < 0.001; children: *Z *= 4.21, *p* < 0.01). Thus, both age groups were able to accurately identify successful from unsuccessful performance.

### 3.3 Did condition affect performance monitoring?

**Figure 3:**
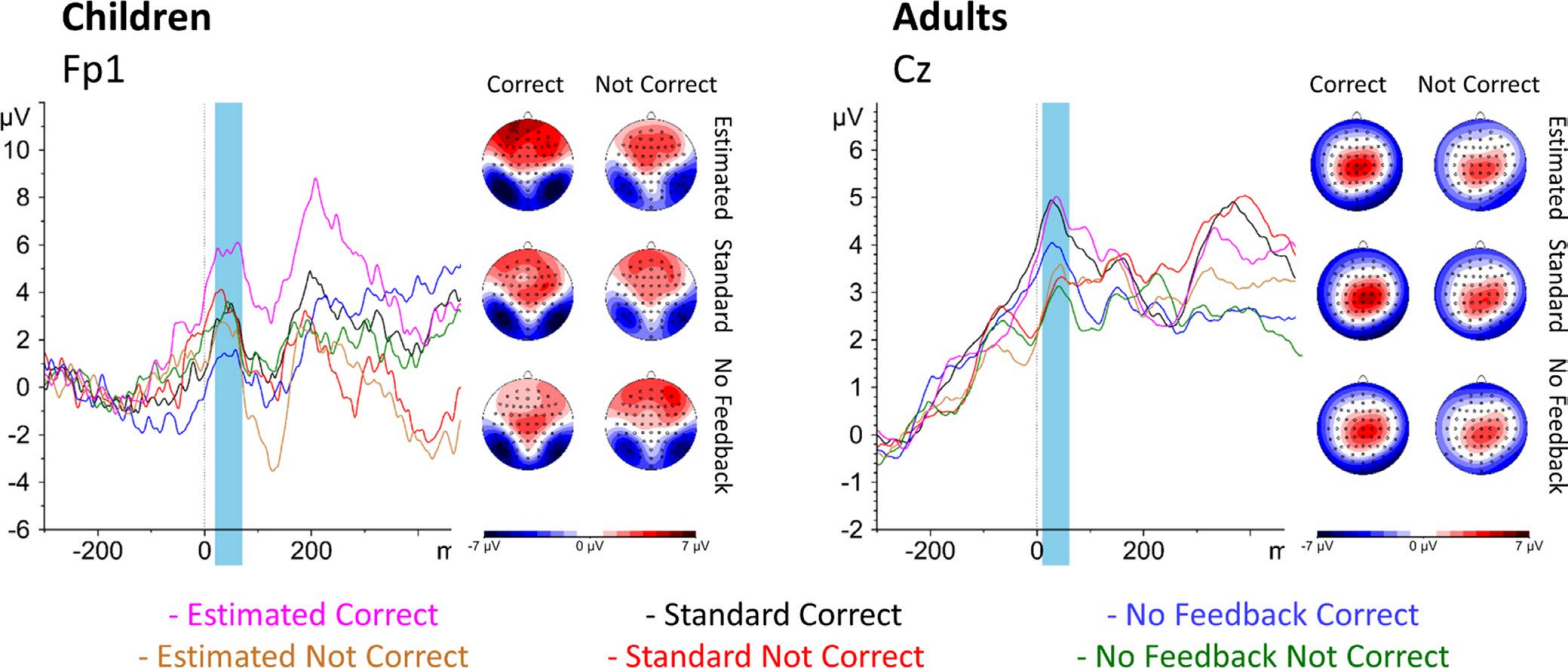
Response‐locked activity by condition and accuracy in children (left panel) and adults (right panel). Topography of the correct response positivity (CRP) is shown beside the waveforms at the peak channels (Cz in adults and Fp1 in children). Blue windows indicate windows used for analysis. Note that scale is different for children and adults. Children showed a more marked CRP for correct relative to not correct responses in the estimated feedback condition only, whereas adults showed a more marked CRP for correct relative to not correct responses in both the estimated and standard feedback conditions

CRP amplitude was predicted by accuracy, *F*(1, 82.49) = 6.19, *p* = 0.015, as well as the interaction between accuracy and condition, *F*(2, 155.93) = 4.67, *p* = 0.012, and the interaction between accuracy, condition, and age, *F*(2, 155.93) = 3.85, *p* = 0.023 (Figure 3). Children showed increased amplitude for correct responses in the estimated feedback condition, *t*(161.80) = 3.60, *p* < 0.001, but not in the standard condition, *t*(161.58) = −0.55, *p* = 0.583, or no feedback condition, *t*(164.29) = −1.46, *p* = 0.145. Adults, however, showed a marginal effect of accuracy in the estimated condition, *t*(158.89) = 1.79, *p* = 0.076, as well as a significant effect of accuracy in the standard condition, *t*(159.41) = 2.01, *p* = 0.046, but no effect in the no feedback condition, *t*(158.89) = 1.26, *p* = 0.209.

### 3.4 Did proactive control engagement vary across conditions?

#### 3.4.1 Flanker‐locked P1

**Figure 4:**
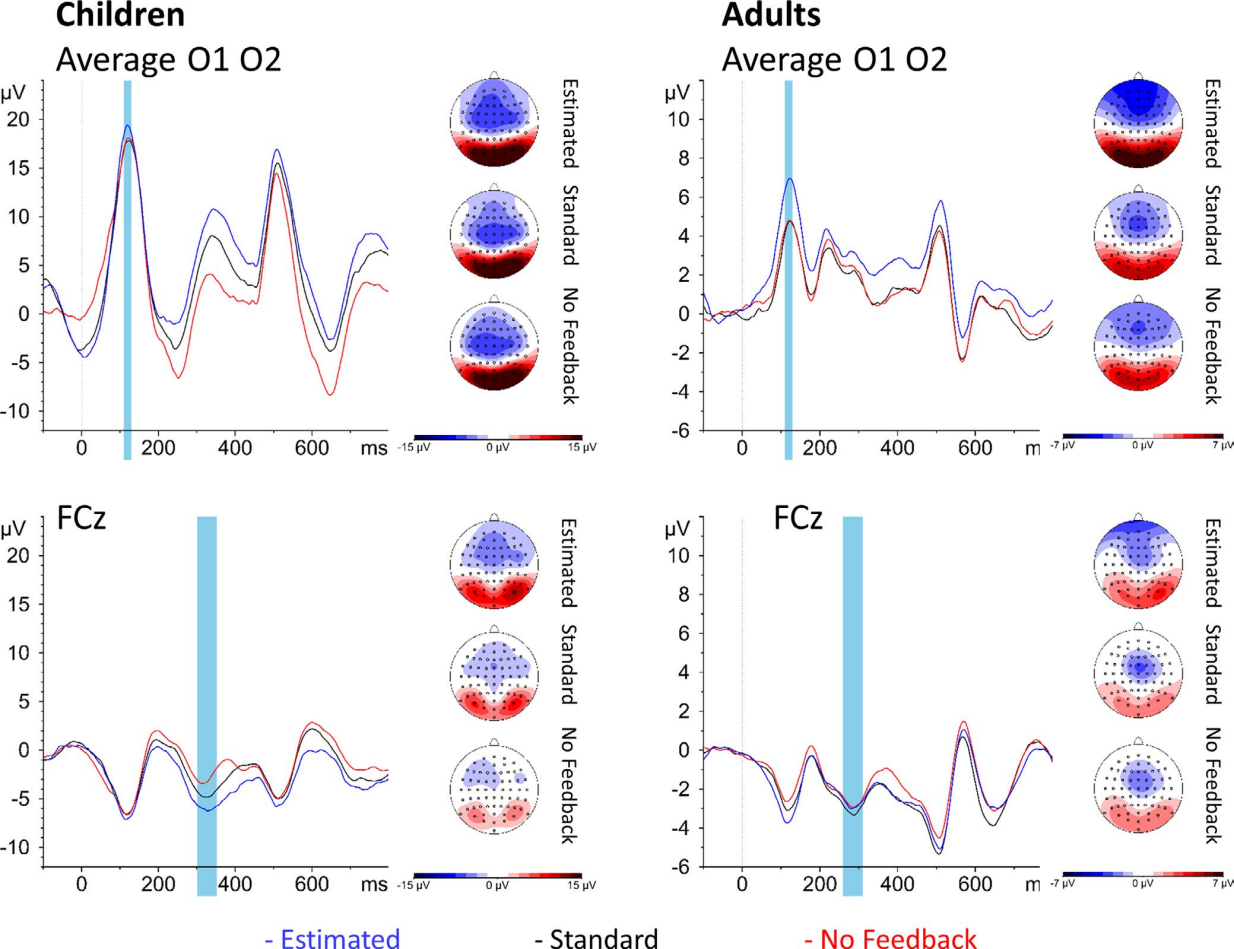
Flanker‐locked activity by condition in children (left panel) and adults (right panel). Upper panel: P1 topography is shown beside the waveforms averaged across O1 and O2. Lower panel: N2 topography is shown beside the waveforms at FCz. Blue windows indicate windows used for analysis. Both children and adults showed a more marked P1 in the estimated condition relative to either other condition, but only children additionally showed a more marked N2 in the estimated feedback condition relative to the no feedback condition

P1 amplitude was greater in children than adults, *F*(1, 54.93) = 86.56, *p* < 0.001 (Figure 4). Furthermore, a condition effect, *F*(2, 107.42) = 3.95, *p *= 0.022, indicated that P1 amplitude was greater in the estimated than either the no feedback condition, *t*(107.54) = 2.41, *p* = 0.046, or the standard feedback condition, *t*(107.46) = 2.46, *p* = 0.041.

#### 3.4.2 Flanker‐locked N2

N2 amplitude was predicted by age group, *F*(1, 50.67) = 4.84, *p* = 0.032, and condition, *F*(2, 103.23) = 4.53, *p* = 0.013. Importantly, an interaction between age group and condition, *F*(2, 103.23) = 3.13, *p* = 0.048, revealed that children showed a greater N2 for the estimated feedback condition than no feedback condition, *t*(108.36) = −3.72, *p* < 0.001, while the standard feedback condition did not differ significantly from either estimated feedback, *t*(107.67) = −1.78, *p* = 0.182, or no feedback, *t*(107.67) = −1.99, *p* = 0.119. Adults, on the other hand, showed no difference between any conditions, *p*s > 0.812.

### 3.5 Did participants engage control reactively when not monitoring performance?

#### 3.5.1 Target‐locked P1

**Figure 5:**
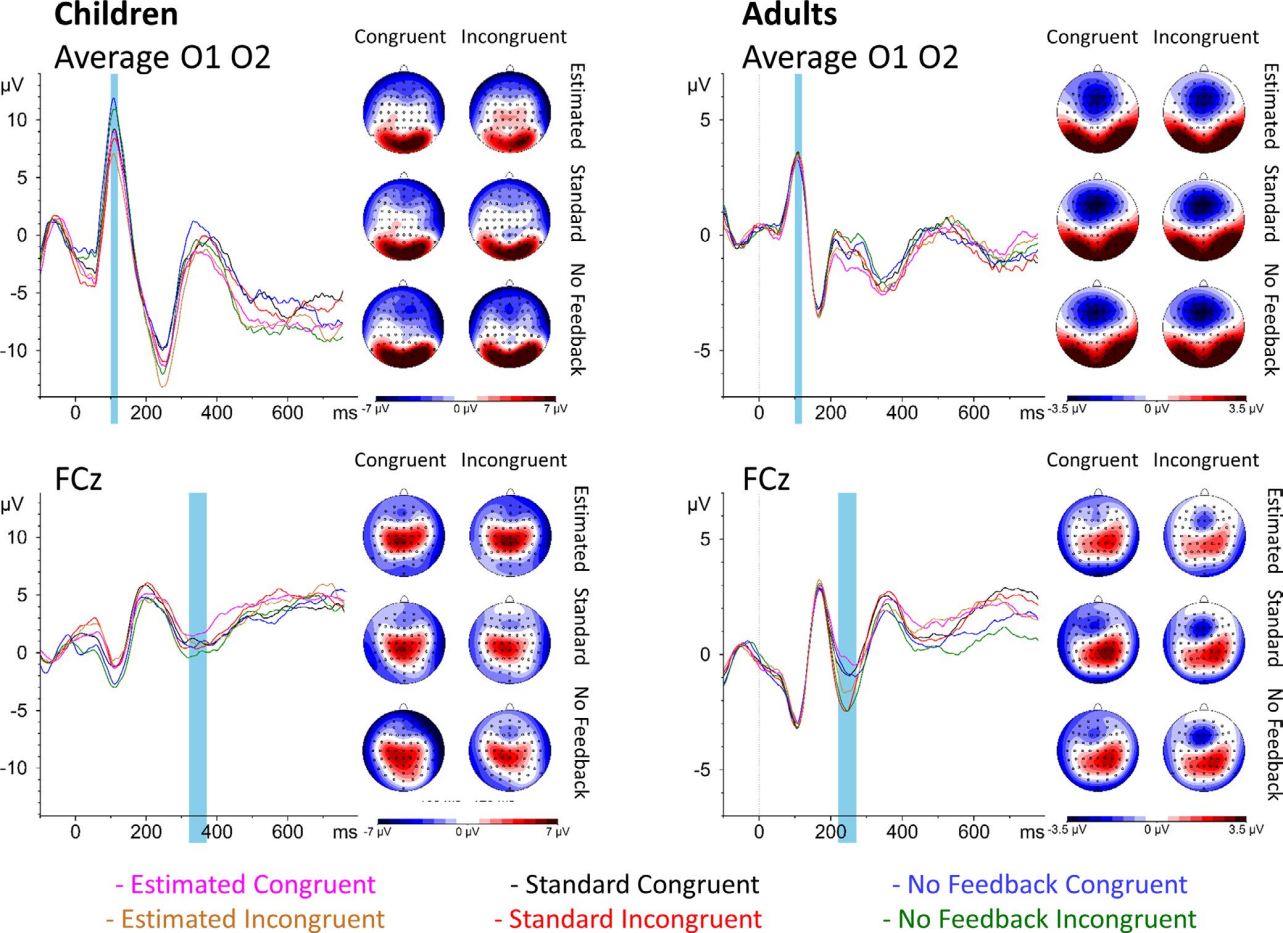
Target‐locked activity by condition in children (left panel) and adults (right panel). Upper panel: P1 topography is shown beside the waveforms averaged across O1 and O2. Lower panel: N2 topography is shown beside the waveforms at FCz. Blue windows indicate windows used for analysis. Children showed a more marked P1 in the no feedback condition relative to either other condition, as well as a more marked P1 in congruent relative to incongruent trials. Across children and adults, the N2 was more marked for incongruent relative to congruent trials

P1 amplitude was predicted by age group, *F*(1, 54.86) = 11.04, *p* = 0.002, and condition, *F*(2, 51.96) = 4.08, *p* = 0.023, as well as an interaction between age group and condition, *F*(2, 51.96) = 5.49, *p* = 0.007 (Figure 5). While adults showed no difference between conditions, *p*s > 0.789, children showed a more pronounced P1 amplitude in the no feedback condition than either the standard feedback condition, *t*(53.31) = −3.47, *p* = 0.003, or the estimated feedback condition, *t*(56.13) = −3.80, *p* = 0.001. Finally, there was an interaction between age group and congruence, *F*(1, 96.99) = 4.35, *p* = 0.040; children showed a more pronounced P1 amplitude for congruent targets compared to incongruent targets, *t*(56.55) = 2.43, *p* = 0.018, while adults showed no congruence effect, *t*(52.77) = −0.44, *p* = 0.662.

#### 3.5.2 Target‐locked N2

N2 amplitude was predicted by age group, *F*(1, 55.02) = 6.38, *p* = 0.014, being more marked in adults than children, and by congruence, *F*(1, 185.38) = 14.97, *p* < 0.001, with a more marked N2 in incongruent than congruent trials. There was no effect of condition, *F*(2, 61.68) = 1.50, *p* = 0.230.

## 4 DISCUSSION

In this study, both children and adults were able to estimate feedback better than chance. Critically, when monitoring was encouraged through feedback estimation, children showed neural evidence of greater performance monitoring as well as increased proactive control. Hence, encouraging children to monitor their performance led to a shift to proactive control that highlights the crucial role of performance monitoring for efficient and flexible control engagement in children.

Children aged 5–7 were able to reflect on their performance to accurately evaluate their responses as correct, incorrect, or late, during a simple flanker task and choose the appropriate feedback. While it has been shown that children's ability to evaluate their responses increases between 7 and 8 years old (Garrett, Mazzocco, & Baker, 2006), our findings show that children can make correct estimates for a simple task even younger, though they do so less accurately than adults. Furthermore, when encouraged to monitor performance, these young children showed neural evidence of performance monitoring through enhanced CRP amplitude in correct (compared to not correct) trials. The lack of CRP effect in the standard feedback condition suggests that mere feedback provision is not enough to encourage children to monitor performance. In that condition, children may have relied on the feedback to recognize their performance success, in line with children's strong reliance on external cues until early adolescence (Crone et al., 2006; Eppinger, Mock, & Kray, 2009). Unlike children, adults showed a pronounced CRP in both standard and estimated feedback conditions, suggesting mere feedback provision was enough for adults to monitor their performance. Indeed, adults likely already monitored performance in the no feedback condition, but the lack of CRP could indicate that the component is sensitive to the salience of response outcomes like other early error‐related components (Hajcak, Moser, Yeung, & Simons, 2005). Importantly, topographical differences in the CRP between groups (being more frontal in children) suggest that while children can be encouraged to monitor performance, their monitoring processes may nonetheless differ to those used by adults. A less developed monitoring mechanism would also be consistent with the children's reduced ability to evaluate their response accuracy in comparison to adults.

Consistent with systematic performance monitoring, adults showed no differences in flanker‐locked N2 across conditions (despite greater flanker‐locked P1 when estimating feedback), probably because they consistently engaged proactive inhibition in all conditions. Such proactive strategies have previously been reported in adults in similar flanker tasks (Giesen et al., 2018). In contrast, feedback estimation critically promoted proactive attention directing and inhibition in children, as evidenced by more pronounced flanker‐locked P1 and N2 in that condition. Interestingly, greater proactive control would be expected to be accompanied by reduced reactive control (see Braver, 2012). Yet, in our study, while the flanker‐ and target‐locked P1 effects in children indicate an efficient shift to proactive control in the estimated feedback condition, the N2 effects suggest the increase in inhibition during the flanker period came with no complementary decrease in inhibition during the target period, relative to the no feedback condition. This pattern raises the intriguing possibility that, instead of shifting from reactive to proactive inhibition in the estimated feedback condition, children employed proactive inhibition *in addition* to reactive inhibition. There are at least two possible accounts for this pattern. First, children of this age may be inexperienced and still inefficient proactive control users, and thus may have needed to compensate by continuing inhibiting flankers after target onset. Second, while young children may indeed be able to use performance monitoring to engage inhibitory control earlier, they may not yet show the corresponding ability to release inhibitory control earlier, suggesting that engaging and disengaging control could be two different processes dependent on different skills.

Regardless, children's longer RTs in the estimated feedback condition than the other two conditions may reflect the increased effort required for such prolonged inhibition. This would be consistent with previous findings showing that encouraging proactive control can lead to a speed‐accuracy trade‐off, such as faster responses at the cost of lower accuracy in 5‐year‐olds (Chevalier et al., 2015). Alternatively, longer RTs in the estimated feedback condition may reflect the additional demand to switch between the target orientation task and feedback estimation in that condition. Specifically, reassigning response button meanings within each trial in this condition may have been especially costly to children given their lower switching abilities than in adults (e.g., Cepeda, Kramer, & Gonzalez de Sather, 2001). However, as greater inhibition often translates into more accurate but slower responses in early childhood (e.g. Wiebe, Sheffield, & Espy, 2012), slower responses with estimated feedback may paradoxically be the behavioural counterpart of more efficient inhibition at that age.

Consistent with prior studies, children did not spontaneously engage proactive control despite being capable of it. Increased proactive control when children were encouraged to monitor performance shows that such suboptimal control engagement stems in part from inefficient monitoring of performance (despite clear ability to monitor performance when encouraged to do so). Topography differences suggest that even when children do monitor performance, they use a different (and potentially less developed) monitoring process to adults, which could impact reliance on such information for control adjustment. Yet inefficient performance monitoring is likely not the only factor contributing to suboptimal control engagement; the emergence of metacognition may also be critical for mature control strategies. Recent findings suggest that children fail to use variations in task demands to modulate engagement of control and related cognitive effort (Niebaum, Chevalier, Guild, & Munakata, 2018; O'Leary & Sloutsky, 2017). For instance, although children can distinguish between easy and difficult tasks, they do not use this information to appropriately allocate more cognitive effort to the difficult tasks until later in childhood (e.g. Destan, Hembacher, Ghetti, & Roebers, 2014; Dufresne & Kobasigawa, 1989; Lockl & Schneider, 2004). Such findings suggest that cognitive control engagement could be efficiently supported by encouraging children to meta‐cognitively reflect on how to best engage control to meet task demands, opening up new intervention perspectives. Indeed 5‐year‐old children show more adult‐like ERP markers on the flanker task after a short training involving such metacognitive reflection (Pozuelos et al., 2018).

Our findings suggest that performance monitoring is critical for mature control engagement. While children engaged control reactively by default, even young children were capable of engaging control proactively when encouraged to monitor performance by estimating their own feedback. Since this shift towards proactive control did not occur when children were presented with passive feedback, metacognitive reflection may be key for efficient cognitive control engagement.

## CONFLICT OF INTEREST

The authors declare no conflict of interest.

## Data Availability

The data that support the findings of this study are available from Lauren V. Hadley upon reasonable request.
